# Reflections on my international genetic counseling rotations: Contrasts in practice between India and the United States

**DOI:** 10.1016/j.gimo.2024.101871

**Published:** 2024-07-20

**Authors:** Niharika Jadeja, Nivedita Rajakumar, Neeraja Reddy, Nadia Ali, Lauren Lichten

**Affiliations:** 1 Department of Human Genetics, Emory University School of Medicine, Atlanta, GA; 2 Department of Molecular Genetics, Neuberg Supratech Reference Laboratories, Ahmedabad, Gujarat, India; 3 Genetic Counseling Division, Mapmygenome India Limited, Hyderabad, India

As a genetic counseling student in the United States, I appreciate the comprehensive nature of my graduate education, which has equipped me with a robust foundation in medical genetics and psychosocial counseling skills. My training has instilled in me the ability to practice with not just cultural competency but also a profound sense of cultural humility, enabling me to approach patient care with sensitivity and respect.

However, it has come to my attention that our educational focus is predominantly centered on the United States, leading to a myopic understanding of genetic counseling practices on a global scale. Although I cannot speak for genetic counseling programs in other countries, I have realized that there may be limited exposure to the diverse practices that exist worldwide. We seem to be operating in relative isolation, with our knowledge contained within separate boundaries. Many of us, me included, lack awareness about the broader international landscape of genetic counseling.

I am originally from India, and I have always felt a passionate commitment to serve the Indian patient population and increase access to high-quality care. With the help of my incredibly supportive program, I had the unique opportunity to return home to complete my summer rotations internationally, in India, which furthered my passion for bridging gaps in the international training and practice of genetic counseling. This unique experience of receiving training in 2 countries allowed me to gain an understanding of some of the similarities and differences in genetic counseling in these 2 nations.

I must emphasize that my brief 3-month rotation experience in India cannot provide a comprehensive understanding of the entire Indian genetic counseling system, just as my rotations in Atlanta, Georgia, cannot offer an all-encompassing view of the entire field in the United States. It would be reductive and oversimplifying to make such generalizations about these diverse and complex health care landscapes. However, my international experience did open my eyes to the profound influence of culture, available resources, and health care systems on the practice of genetic counseling, fostering a deeper appreciation for the rich diversity that characterizes our profession on a global scale.

That being said, the following article is a reflection of the similarities and differences in genetic counseling practice in India and the United States that I noticed as part of my training. I would first like to frame this discussion by acknowledging the context of my experiences. I completed 2 rotations (200 hours and 50 hours, respectively) in India. Both of these rotations were conducted in a private lab setting, which may limit the generalizability of my observations. In the United States, my genetic counseling experience was primarily in the outpatient setting in a large academic medical center (4 200 hour, and 2 50 hour rotations). These settings are important to contextualize my experiences and prevent generalizing them to larger contexts than appropriate. Second, it is important to state that this article summarizes my own experiences and observations, with some literature to back up the experiences or help with interpretation of the situations. The discussion is not meant to generalize the practices, nor language, cultural, or religious beliefs in either country.

### Genetic testing accessibility and strategies

One of the differences between my experiences in India and the United States relates to genetic testing strategies, which arise from underlying differences in the health care systems. It is important to recognize that health care coverage in India varies widely, with government schemes offering different levels of coverage from state to state. Because my rotations were in a private lab setting, we were primarily working with private insurance companies, which often classified genetic testing, regardless of its nature, as elective, leaving patients responsible for the costs. In the United States, with my genetic counseling experience primarily in the outpatient setting, the cost of genetic testing was intricately linked to patients’ insurance coverage. Unlike my experiences with insurance in India, where the private insurance companies we worked with did not cover genetic tests, and patients covered their testing costs, my experience with insurance in the United States was far from standardized. Here, the availability and coverage of genetic testing varied widely depending on the individual’s insurance plan. A significant aspect of our role as genetic counselors in the US entails engaging in dialog with insurance companies to advocate for our patients’ essential care, which was not the case in India, since the question of insurance coverage often did not arise here.

These financial considerations profoundly influence testing strategies. In a private lab setting in India, the preferred approach was often to use exome sequencing as a first-tier test for conditions in which molecular testing would be recommended. This strategy offers the highest diagnostic yield while minimizing costs for the patients, as opposed to the conventional practice of sequentially ordering gene panels followed by an exome that I observed in the United States. This sequential testing strategy risks the patient covering the cost of multiple rounds of testing, for an answer that could be identified on a single test. This approach does, however, introduce complexities related to secondary findings and questions of coverage in test methodology, which affects the likelihood of obtaining a diagnosis.^1^ In this regard, as the United States considers the incorporation of exomes as a first-tier test, it can look to countries such as India where it is already used in this manner.

Another testing strategy that I observed during my rotation was that, at least in the private lab setting, financial considerations for patients in India often led to opting for a proband-only exome rather than a trio exome. When a variant was detected through testing, subsequent targeted parental testing was initiated using Sanger sequencing. Although this approach is more cost-effective than ordering 3 exomes, it introduces complexities in result disclosure, because we do not know if an identified variant is de novo or inherited until follow-up testing is ordered. This introduces concerns about potential loss to follow-up because unresolved questions persist without ongoing patient involvement.

It is important to note that this discussion is somewhat reductive because it does not account for testing options available in government institutions in India or safety net hospitals in the United States, where testing can be free for patients. These institutions play a crucial role in providing access to care for underserved populations.

Of note, one of my rotation experiences in India involved collaborating with the Tamil Nadu state government in its efforts to incorporate genetic testing within the framework of its comprehensive health insurance program. The Chief Ministers’ Comprehensive Health Insurance, by the Government of Tamil Nadu was created with the goal of providing comprehensive health care to the residents of the state, and it includes genetic testing services for individuals of low socioeconomic status, when medically indicated.^2^ My rotation was at a lab that was collaborating with the state government to provide genetic testing that would be covered under this scheme. This collaboration highlights the efforts being made to improve access to genetic testing and health care services for individuals who may not otherwise be able to afford them. It also underscores the importance of public-private partnerships in expanding access to health care and reducing health inequities. I had the opportunity to work alongside genetic counselors at the lab, where they reviewed clinical histories and test details to ensure the appropriateness of tests. This experience provided me with valuable insights into the role of genetic counselors in this laboratory, particularly in determining appropriate test methodologies and increasing accessibility of genetic services in the Indian patient population. The presence of this comprehensive health insurance scheme is a promising step toward improved access to genetic medicine, offering hope for a future in which genetic testing is not confined to individuals of high socioeconomic status.

Similarly, in the United States, I had the opportunity to work at a safety net hospital, where the genetic counselor had acquired grant funding to cover the cost of testing for patients in need of financial assistance, who met criteria for testing. Working closely at both of these rotations allowed me to learn more about different strategies to increase accessibility of genetic testing in these 2 different countries.

### Frequency of uncertain results

In the previous section, we discussed practice differences that I had observed between India and the United States based on differences in the structure of the health care system. Now, we will explore a difference that stems from racial inequities in research. It is widely recognized in the field of genetics that our current reference genome is predominantly built upon individuals of European descent. This often results in a higher prevalence of variants of uncertain significance (VUS) among people of color. Studies across specialties have found the VUS frequency to be higher in non-White than White patients.^3^, ^4^, ^5^, ^6^, ^7^, ^8^, ^9^ The burden of uncertain variants falls on both the patients and the providers, and this experience has been previously investigated in the literature. Patients report feelings of anxiety, worry, distress, and confusion upon receiving VUS results.^10^, ^11^, ^12^, ^13^, ^14^, ^15^ The conceptual, methodological, and ethical problems that complicate VUS disclosure have also been characterized, with providers experiencing challenges with effectively communicating uncertainty in clinical practice, concerns about patient autonomy, and questions regarding net benefits and harms of genetic testing, within the context of uncertain results.^16^

The challenges to VUS disclosure are heightened in countries where the majority population is not White because of the increased frequency of these results. I witnessed the challenges, faced both by patients and providers firsthand during my rotations in India. Comprehension of uncertain results was difficult for patients, especially those with low literacy. We often think about genetic testing as an option to provide patients with an answer, without considering the questions it might add in the case of uncertain results, and I watched several patients grapple with confusion and a lack of clarity about the meaning of their results.

Additionally, there were implications about these findings and their impact on medical management. The approach to managing uncertain results varies depending on the specialty, even within 1 country. In a general genetics setting, a VUS might raise suspicion if the patient’s symptom presentation matches the clinical spectrum of pathogenic variants in the gene. Hence, there were not many differences in how VUS were clinically managed in a general setting while comparing the 2 countries. However, in a cancer genetics setting, results are often categorized into those that change medical management (pathogenic changes) and those that do not (VUS and negative results). Given the higher prevalence of VUS in people of color because of the lack of research into these populations, presence of a VUS does not necessarily lower our suspicion about the pathogenicity of a variant. I noticed this playing a role in my cancer genetic counseling appointments in India. The providers I worked with were more hesitant about dismissing the implications of a VUS, whereas in the United States, my supervisors in cancer often disclosed VUS similar to the manner in which they disclosed negative results. Although providers I worked with in India did not change prophylactic surgery recommendations on the basis of uncertain results, they did recommend further clinical follow-up in patients, to achieve a better understanding of the variant. For example, I attended an appointment in which a patient was found to have a VUS in APC, a gene associated with familial adenomatous polyposis. The primary purpose of testing for this patient was frequent lipomas, unrelated to polyps. However, the genetic counselor I was with for this appointment recommended that the patient receive an eye exam to assess for congenital hypertrophy of the retinal pigment epithelium (a benign, pigmented fundus lesion) and a dental exam for dental anomalies, both of which are frequently observed in familial adenomatous polyposis in addition to colon polyps. This additional information would help make a more informed decision about the pathogenicity of the variant and the amount of surveillance to be recommended. This is not a recommendation that would have been made in my cancer rotations in the United States; therefore, it was interesting to learn of the impact of frequency of uncertain results on clinical management and patient care in India.

In addition to this, providers that I worked with in India put more effort into independent variant interpretation, because they were less able to rely on reported classifications. However, for both of these considerations (clinical recommendations and variant interpretation), it is important to note that I was working with genetics providers. Research has found that a substantial proportion of nongenetics physicians lack an understanding of the implications of VUS, which can have an impact on patient care after genetic testing in both countries.^17^

The elevated frequency of VUS among non-White patients underscores the critical need for ongoing research and efforts to ensure equitable care for patients across the globe. Addressing this disparity is essential to provide comprehensive genetic counseling and medical care for all individuals. Initiatives such as the All of Us Research Program are an excellent way to diversify health databases, and more worldwide programs such as these are necessary to have a more equitable impact.^18^ In addition to All of Us, there are Indian databases that host information about genotype-phenotype associations in the Indian population.^19^, ^20^, ^21^, ^22^, ^23^ Although there are limitations for these resources, their presence is a good starting point to reduce the frequency of VUS in an Indian context.^24^

### Public health screening initiatives

The previous section included a discussion of health inequities and implications; we will now discuss public health screening programs in India and the United States. It is interesting to observe how public health screening guidelines are influenced by the priorities and experiences with disease within each country. In the United States, the disease burden of communicable diseases, measured in disability-adjusted life years, has been relatively low since the 1990s. In 1990, communicable diseases accounted for 7.51% of the disease burden in the United States, and this has further decreased to 4.47% since 2019.^25^ In contrast, India has seen a significant shift in its communicable disease burden, which decreased from 62% to 30.61% between 1990 and 2019.^25^ Noncommunicable diseases (NCDs) have been a priority in the United States for decades; however, the importance of addressing NCDs has only recently gained prominence in India.

This contrast in disease burden is reflected in the screening guidelines for NCDs such as cancer. In the United States, there are established guidelines for breast, cervical, prostate, and colon cancers, with recommendations based on age. Screening for these cancers has been recommended since the 1970s, although specific recommendations and the availability of screening tests have evolved over time. In contrast, India’s national guidelines recommend screening for oral, breast, and cervical cancers alone.^26^ In addition to this, India faces challenges with compliance to screening guidelines,^27^^,^^28^ The general population screening guidelines are important to consider in a cancer genetics setting, as we think of implications of pathogenic variants, which would affect recommended surveillance in comparison with the general population. The shift in burden of and consequent focus on NCDs in India suggests that the focus on cancer screenings will continue to grow, emphasizing the importance of integrating genetic counseling services to address the evolving landscape of preventive health care.

The increase in the burden of NCDs in India has led to the implementation of various public health programs aimed at addressing different aspects of health care. One such program is the Rashtriya Bal Swasthya Karyakram (RBSK), which is a comprehensive initiative designed to screen children for a range of health conditions, including birth defects, developmental delay, nutrient deficiencies, and other childhood diseases.^29^ The program aims to identify health concerns early in childhood, so that appropriate interventions and treatments can be provided to improve health outcomes and quality of life for children across India. RBSK operates at both the national and state levels and is an essential component of India’s efforts to promote child health and well-being. Although the screening guidelines of RBSK are a commendable step toward improving the health and well-being of the Indian population, they do not currently include screening for metabolic or genetic conditions. Implementing a comprehensive newborn screening program in India would be a crucial next step because it could help identify and address these conditions early in life, potentially preventing or minimizing long-term health complications. In the United States, newborn screening is a state-based public health program, in which states screen for some or all of the conditions on the Recommended Uniform Screening Panel.^30^ Medical professionals, public health advocates, and experts in India have been calling for the necessity of newborn screening at a national level as early as the 1980s, In addition to the benefits of implementing newborn screening initiatives worldwide, India stands to gain more from such a program because our population is likely to be disproportionately affected by inborn errors of metabolism, which typically follow an autosomal recessive pattern of inheritance because of higher rates of consanguinity. The prevalence of consanguinity in India is 13.6%, although it ranges significantly across the country, depending on factors including religion, caste, and socioeconomic status.^31^ For example, the prevalence ranged from 3.1% in the North-East to 23% in the South.^32^ Private hospitals and labs in India offer newborn screening to their patients, and learning from systems that are in place will be helpful for the development of public screening programs in the future.^16^ Although the large population in India and the high initial investment serve as challenges to the implementation of a nation-wide program, promising progress is already evident on a state-by-state basis, with Chandigarh, Goa, and Kerala setting exemplary standards for newborn screening in the country.^33^

The presence of different public health initiatives in India and the United States is shaped by their unique experiences with health and disease, as well as the prevalence of health concerns within each population. As India’s proportion of NCDs continues to grow, there will likely be a greater emphasis on further developing and expanding screening programs to improve the overall health of the population. This shift in focus reflects the evolving health care needs and priorities of the Indian population, highlighting the importance of tailored public health interventions to address specific health challenges in different regions and populations.

### Accessibility of pregnancy termination options

Aside from the previous aspects discussed, legal factors also play a significant role in shaping genetic counseling practice and the accessibility of care options in India and the United States, particularly regarding pregnancy termination options. In the United States, there have been notable changes in the accessibility of reproductive choices in recent years, although the landscape varies by state.^34^ As a student in Georgia, where a 6-week ban on abortions is in effect,^35^ I have witnessed the impact of these restrictions. I have been involved in assisting parents who have received challenging news about a pregnancy, individuals who are already grappling with immense emotional distress, without having to contemplate traveling to another state to access the medical care they need. In contrast, India’s legal framework provides abortion options to patients up to 24 weeks gestation, significantly expanding the spectrum of reproductive choices available to them.^36^ This extended time frame offers patients the opportunity to make fully informed decisions without the added pressures of legal concerns. As a result of this, conversations about decision-making during pregnancy in India that I was a part of differed significantly from those that I observed in the United States. In India, the decision to continue or terminate a pregnancy was personal, influenced by individual choices, beliefs, and religious factors, rather than concerns about the accessibility or legality of options for care. It is important to note, however, that the accessibility of care varies widely across the country, and although there are fewer legal restrictions, other factors influence accessibility. For example, previous studies from India have found that women felt discouraged to seek abortion care from public hospitals because of the requirement of repeated visits, longer waiting durations, and perception of poor preservation of confidentiality.^37^, ^38^, ^39^

### Quality of care in different languages

There are also cultural factors that contribute to differences in these countries, and one such factor is the number of languages that are spoken in India and the United States. This influences the quality of care that is provided in appointments to patients who speak languages other than English. India is a multilingual country, with 22 official languages; therefore, in my first rotation in India, patients would specify their preferred language while scheduling an appointment, ensuring that they could consult with a genetic counselor fluent in that language. In the United States, appointments are primarily conducted in English, and health care interpreters are used to mediate appointments with patients who do not speak English. The population of US residents who do not speak English as their primary language has significantly increased in recent decades. In 2012, US residents speaking a language other than English at home represented 1 in 5 individuals, standing at 64.7 million people, having nearly tripled since 1980. These numbers have continued to rise in recent years and represent a substantial portion of our patient population.^40^ Although strides are being made in the United States to embrace more inclusive practices for patients with limited English proficiency, the availability of interpreters remains limited. It is also important to acknowledge that appointments involving interpreters understandably take twice as long, and logistical challenges can arise when a patient and interpreter speak different dialects of the same language, resulting in potential miscommunication.

It was interesting to observe appointments in Indian languages during my rotations in India. I had a grasp of some languages, and it was helpful to understand how fluent speakers would frame a counseling appointment in that language. I also observed appointments in languages I did not understand, and it was impactful to see patients being able to communicate in their language of comfort to speak with a provider. It is important to note that my rotation in India was in a private lab setting; therefore, the emphasis on patient language preference is unlikely to be applicable to all hospitals and institutions across the country, particularly those with limited staff proficient in multiple languages. Nevertheless, this is a quality that I hope the United States continues to develop as its non-English speaking patient population continues to grow. Additionally, India’s multilingual environment poses both advantages and challenges. On one hand, patients are more likely to find a health care provider who speaks their language, enhancing communication and rapport. On the other hand, the scarcity of interpreters can hinder effective communication, raising crucial questions about obtaining informed consent when patients and providers do not share a common language. This underscores the importance of addressing language barriers in health care to ensure that all patients receive equitable and comprehensive care.

### Views on disability

Another cultural factor to consider is the perception of disability, especially because in genetics, a significant portion of the patient population that we serve includes disabled people. During my training in the United States, I have had the privilege of working closely with the disability community, and this experience has been incredibly enlightening. These experiences have taught me to prioritize patient autonomy, experience, and priorities. I also learned from my clinical experiences to provide patients with a wide array of resources, including support groups for their disabilities and referrals to physical therapy, occupational therapy, speech therapy, and individualized education programs and accommodations in schools. These resources are present in India, but their accessibility can be constrained and often limited to patients and families of higher socioeconomic status.

Aside from resources we can provide to patients, it is imperative to assess whether the care we offer is free from ableism, especially considering the historical ties of the genetics field to eugenics. During my rotations in India, I did encounter instances of ableist language, such as suggesting “prevention of” consanguineous marriage to reduce the chance of having children with disabilities. In 2019, the Medical Council of India released a Competency-based Curriculum for Indian medical undergraduates, and the curriculum did not adequately represent disability care, using outdated terminology and propagated misconceptions, including the polarization of “normal life” opposed to disabled life. In response to this, doctors with disabilities, disabilities rights activists, and health professions educators developed competencies for disability care education in medical training.^41^ I view this as a promising step to improved quality of care provided to patients with disabilities in India.

It is worth noting that the quality of care for disabled individuals in the United States also has room for improvement, and instances of ableism among providers are not uncommon. My rotations in the United States were in a large academic medical center; therefore, although I did not personally witness ableism that stood out to me, my experiences are not generalizable to hospitals and clinics throughout the country.

### Balance of privacy and care

Another cultural contrast between genetic counseling practices in the United States and India lies in the approach to privacy and confidentiality. In the United States, strict regulations such as the Health Insurance Portability and Accountability Act protect patient health information, ensuring its privacy. Although these safeguards are crucial, they can sometimes limit the exchange of pertinent genetic information among family members, hindering comprehensive care. For example, a classmate of mine shared that they had an appointment with a family member of a patient that their supervisor had previously seen. Neither the original patient nor the family member were aware that they both received care from the same genetic counselor, because they both sought care for different reasons. The genetic counselor was aware that the first patient had Von Hippel Lindau Syndrome, but because they personally had not shared this information with the family member, the genetic counselor was unable to disclose this, despite being aware that the family member was at risk for tumors and would benefit from the recommended screening guidelines. The balance between privacy and care is crucial, and case studies such as this highlight how these principles can sometimes be at odds with each other.

In India, on the other hand, health care data security and privacy protections are less stringent, and the focus tends to prioritize the patient's health over privacy concerns. Although this approach can facilitate more open discussions about genetic risks within families, it also raises concerns about data security and insurance discrimination based on genetic information. The absence of legislation similar to the Genetic Information Non-Discrimination Act in the United States leaves individuals in India vulnerable to potential discrimination based on genetic testing results, which plays a role in decision making for testing. Despite these drawbacks, the patient-centered approach in Indian genetic counseling emphasizes immediate health needs over privacy concerns, highlighting a different set of priorities in care delivery.

As I reflect on my experiences in genetic counseling across the United States and India, I am struck by the similarities and differences in approach to genetic counseling and the valuable insights they offer. Navigating these 2 worlds has broadened my perspective and deepened my interest in bridging the gaps in genetic counseling practice worldwide. From my clinical experience in the United States, I learned the importance of cultural humility and patient autonomy and privacy and also gained an appreciation for the public health screening programs in place. From my rotations in India, I learned about the diversity of cultural beliefs, gained an understanding of counseling in a multilingual country, and also acquired valuable experience with variant interpretation in a majority non-White population. Despite the differences in practice, both countries share a common goal: to provide quality care to their populations. I believe there is much to learn from each other, and I hope that by sharing these experiences, we can work toward a more inclusive and accessible future for genetic counseling across the globe. I hope that more students have opportunities such as this in the future to improve their own and our collective understanding of genetic counseling across the globe.

## Declaration of AI and AI-Assisted Technologies in the Writing Process

During the preparation of this work the author used ChatGPT to enhance the flow and readability of the content. After using this tool/service, the author reviewed and edited the content as needed and takes full responsibility for the content of the publication.

## Conflict of Interest

I, Niharika Jadeja, am a graduate student currently enrolled at Emory University, and am the first author of this manuscript. Emory University School of Medicine facilitated my involvement in this project by permitting me to undertake the international summer rotations. Additionally, 2 of the co-authors, Nadia Ali and Lauren Lichten, are faculty members affiliated with the Emory Genetic Counseling Training Program.

During the rotations, my supervisors were Neeraja Reddy and Nivedita Rajakumar. Both genetic counselors are employed by private genetic testing companies, MapMyGenome and Neuberg Supratech.

I affirm that these affiliations did not unduly influence this article.

I am committed to addressing any inquiries or concerns related to these potential conflicts of interest and providing additional information if necessary.
